# Evaluation of Antimicrobial and Cytotoxic Activity of Nanoformulated Chamomile and Green Tea-Based Mouthwash: An In Vitro Study

**DOI:** 10.7759/cureus.57470

**Published:** 2024-04-02

**Authors:** Shubhangini Chatterjee, Jaiganesh Ramamurthy

**Affiliations:** 1 Department of Periodontics, Saveetha Dental College and Hospital, Saveetha Institute of Medical and Technical Sciences, Saveetha University, Chennai, IND

**Keywords:** nauplii, brine shrimps, cytotoxic, antimicrobial, mouthwash, green synthesis, green tea, chamomile tea, zinc oxide, nanoparticles

## Abstract

Introduction

Nanotechnology plays a significant role in the biomedical and dental fields, offering numerous benefits to humans. Particularly, nanoparticles synthesised through green methods involving herbal formulations present promising advantages. Zinc oxide nanoparticles (ZnONPs) demonstrate strong antibacterial properties. Utilising treatments incorporating chamomile tea and green tea may potentially reduce toxicity while enhancing antibacterial effectiveness against oral infections. This study aimed to develop a mouthwash containing ZnONPs, followed by an evaluation of both its cytotoxicity and antibacterial effectiveness.

Materials and methods

This study was conducted at Saveetha Dental College, Saveetha Institute of Medical and Technical Science, Chennai, India. In the synthesis of ZnONPs, a formulation consisting of chamomile tea and green tea was employed. Subsequently, these synthesised nanoparticles were used in the preparation of mouthwash. An antimicrobial test of the produced ZnONPs was carried out using the agar well diffusion technique for oral pathogens. For analysis of cytotoxicity, brine shrimps were used in an assay, and comparisons were made with a commercially available mouthwash.

Results

The antimicrobial properties were assessed, and the formulated mouthwash demonstrated a zone of inhibition of *Staphylococcus aureus *(20 mm)*, Enterococcus faecalis *(11 mm)*, Streptococcus mutans *(15 mm)* *and *Candida albicans *(13 mm), when the agar well diffusion assay was carried out. Furthermore, the formulated mouthwash exhibited lower cytotoxicity compared to the commercially available mouthwash when cytotoxicity was checked in brine shrimps.

Conclusion

In our study, the ZnONP synthesis with chamomile tea and green tea showed notable antibacterial and antifungal effects. In addition, lower toxicity was observed compared to the commercially available mouthwash. These findings suggest that mouthwash formulated with green-synthesis ZnONPs could serve as a viable alternative to synthetic mouthwash options. As a result, it is suggested that ZnONPs could be employed in mouthwash formulations at concentrations of 40 µL.

## Introduction

Nanotechnology represents a burgeoning field with diverse uses across varied sectors. Its significance is evident in drug distribution, diagnostic methods, antibacterial therapies, wound care and the management of ailments, such as cancer, cardiovascular issues, diabetes and kidney diseases [1]. Utilizing nanoparticles derived from plant-based green synthesis is an emerging approach, the medicinal advantages of which are still under exploration. The advancement of nanobiotechnology in medical applications has introduced the concept of regulating and addressing human biological systems via nano-formulated materials [2].

Zinc oxide nanoparticles (ZnONPs), widely acknowledged as being less toxic, are often preferred over alternative metal oxide ones. ZnONPs exhibit numerous biomedical uses, including antibacterial, anti-inflammatory, antidiabetic and anticancer effects. In addition, they find applications in wastewater treatment to eliminate impurities, pollutants and certain metals through the process of photodegradation [3]. ZnONPs are known for their effective antibacterial properties against a variety of bacterial strains [4]. The reduced size of ZnONPs contributes to their potential antibacterial efficacy by enabling efficient penetration into cells to eradicate microorganisms. Moreover, ZnONPs are used widely for the fabrication of a variety of dental products [5].

Nanoparticles can be synthesised through two prevalent methods: chemical synthesis and green synthesis. The green synthesis method stands out for its eco-friendliness, cost-effectiveness and efficiency. Furthermore, a multitude of plants, seaweeds and marine life harbour a variety of compounds, such as polysaccharides, pigmented materials and phenols. 

Bacteria and fungi, collectively known as oral pathogens, have an important role in the development of various oral ailments - commonly periodontal diseases and caries [6]. Due to an increase in the number of drug-resistant pathogens, there is an urgent requirement to investigate alternatives, which can effectively act against such oral pathogens [7].

Combining certain herbal-derived formulations and nanoparticles offers a promising alternative to enhance their effectiveness [8]. The compounds found in medicinal plants and their combination with certain properties of nanoparticles have the potential to produce more powerful antimicrobial agents. Specifically, by incorporating ZnONPs into herbal formulations of plants like chamomile tea and green tea, there is the prospect of boosting their antimicrobial effectiveness against oral pathogens [9]. Both chamomile tea and green tea offer promising avenues for harnessing natural antimicrobial agents, providing valuable alternatives in combating microbial infections. By combining these two, a synergistic effect can be harnessed [9]. Chamomile tea contains bioactive compounds, i.e., flavonoids and terpenoids, which have notable antimicrobial effects against numerous pathogens [10]. In addition, studies have demonstrated the inhibitory effects of chamomile extracts on bacterial growth, indicating their potential as a natural antimicrobial agent [11]. Green tea is rich in polyphenolic compounds, particularly catechins, like epigallocatechin gallate (EGCG), which have been extensively studied for their antimicrobial properties [12].

This study aimed to assess the potential of a nanoformulated chamomile tea and green tea extract against oral pathogens. This method is both economical and environmentally friendly, characterised by reduced energy usage and minimal technical skill and equipment requirements. The assessment involved measuring the inhibition zone; for this, an agar well diffusion assay was employed. In addition, in order to assess the cytotoxic effects of the formulated mouthwash containing ZnONPs, brine shrimps were used.

## Materials and methods

This study was conducted at Saveetha Dental College, Saveetha Institute of Medical and Technical Science, Chennai, India.

ZnONP preparation

The green synthesis of ZnONPs was done in accordance with Chatterjee et al. (2023) [9]. Initially, the chamomile (Twinings) and green (Tetley) tea leaves underwent a thorough washing in distilled water to ensure cleanliness. Following this, the leaves were finely ground into particles using a mortar and pestle, with their respective weights carefully measured. A blend of green and chamomile tea leaves was then prepared to achieve a total weight of 1 gram. Conical flasks were subsequently filled with 100 ml of distilled water, into which the accurately measured tea leaf mixtures were introduced to aid in dissolution.

A one-molar solution of the tea extract was created by meticulously mixing the blend and subjecting it to heating at 60 degrees Celsius for 15 to 20 minutes, followed by filtration. The resulting extracts from chamomile and green tea were combined with 0.016 g of zinc oxide and 90 ml of distilled water. Regular observations were conducted to monitor any colour changes in the solution, which were documented through photography and recording [9].

Formulation of mouthwash

It was formulated with the ingredients as depicted in Table 1. 

**Table 1:** Constituents of the herbal formulated mouthwash

Materials	Functions
9.5 mL distilled water	Solvent
500 μg nanoformulated extract	Main ingredient
0.3 g sucrose	Sweetener
0.001 g sodium benzoate	Preservative
0.01 g sodium lauryl sulphate	Foaming agent
0.1 mL peppermint oil	Flavouring agent

Following this, 500 g/mL of the nanoformulated extract was introduced into the mouthwash mixture, and ultimately, 10 mL was prepared for further experimentation [13].

Antimicrobial test

The Kirby-Bauer test was employed to assess the antimicrobial activity. Muller-Hinton agar was utilised for the culture of bacteria, while Rose Bengal agar was employed to test for fungi. Sterile cotton swabs were used to spread isolates onto the respective agar plates. A sterile polystyrene tip was used in order to create a 9-mm-diameter well on the agar plate. Subsequently, different concentrations of nanoformulated mouthwash from 25 to 100 g/mL were added. Amoxyrite and fluconazole were used as the standard for bacterial and fungal cultures, respectively. Following the addition of antimicrobial agents to the wells, Petri plates were kept for one day and two days, respectively, for the incubation of bacterial and fungal cultures. This was done at a temperature of 37 degrees Celsius. After the incubation period, the zone of inhibition diameter measurements was made around each well.

Cytotoxicity test

Two grams of iodine-free salt were added to 200 mL distilled water, and the mixture was thoroughly mixed. A six-well enzyme-linked immunosorbent assay (ELISA) plate reader was prepared by adding 10-12 mL of salt water to each well to which 10 nauplii were added. Concentrations ranging from 5 to 80 g/mL of the mouthwash were then put into each well. The control well had salt water, and live nauplii were recorded as per previous studies [14]. 

One-day incubation of the ELISA plate was done followed by the analysis of a number of live nauplii. The percentage of live nauplii was calculated for the brine shrimp lethality assay as follows:

Percentage of live nauplii = Number of dead nauplii / (Number of dead nauplii + Number of live nauplii) x 100

Statistical analysis

The data were initially recorded using Microsoft Excel (Microsoft Corporation, USA) and later imported into IBM SPSS Statistics for Windows, version 22.0 (released 2013, IBM Corp., Armonk, NY). Specifically, an independent t-test was conducted for the comparison of the nanoformulated mouthwash and the control at various concentrations.

## Results

Antimicrobial activity

Analysis was done for *Staphylococcus aureus, Streptococcus mutans, Enterococcus faecalis and Candida albicans*. Amoxicillin and fluconazole were employed as the standard for bacterial and fungal cultures, respectively. At 100 g/mL, ZnONPs demonstrated considerable antimicrobial activity, the zone of inhibition was as follows: *S. aureus* (20 mm), *E. faecalis* (11 mm), *S. mutans* (15 mm) and *C. albicans* (13 mm), as depicted in Table 2.

**Table 2:** Antimicrobial test zone of the inhibition test was measured in millimetres at different concentrations (in microliters)

Microorganism	Zone of inhibition at 25 ul (in mm)	Zone of inhibition at 50 ul (in mm)	Zone of inhibition at 100 ul (in mm)	Zone of inhibition (control antibiotic) (in mm)
Streptococcus aureus	10	10	20	40
Enterococcus faecalis	9	9	11	35
Streptococcus mutans	10	10	15	38
Candida albicans	12	12	13	9

**Figure 1:**
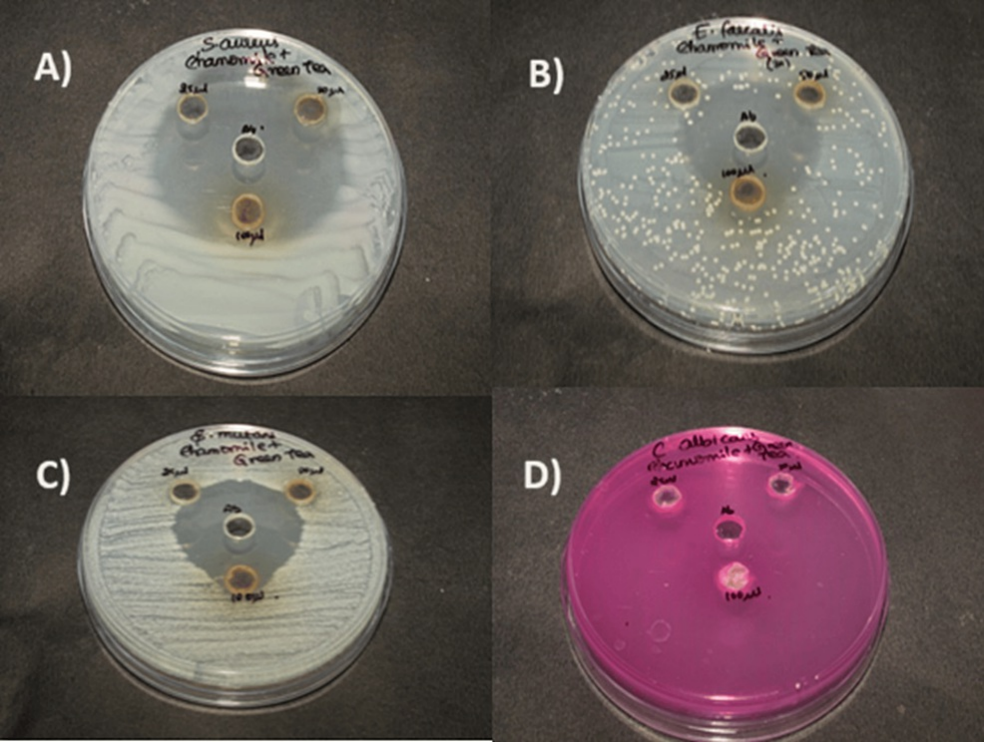
Antimicrobial activity of green synthesised zinc oxide nanoparticles against oral pathogens: a) Streptococcus aureus, b) Enterococcus faecalis, c) Streptococcus mutans, d) Candida albicans. The zinc oxide nanoparticles (ZnONPs) were tested at three different concentrations of 25, 50 and 100 µg/mL. Zone of inhibition depicted at 25 ul, 50 ul and 100 ul, along with that for Ab = antibiotic (control).

These results indicate that chamomile and green tea-mediated ZnONPs have antimicrobial properties, as illustrated in Figure 1.

**Figure 2:**
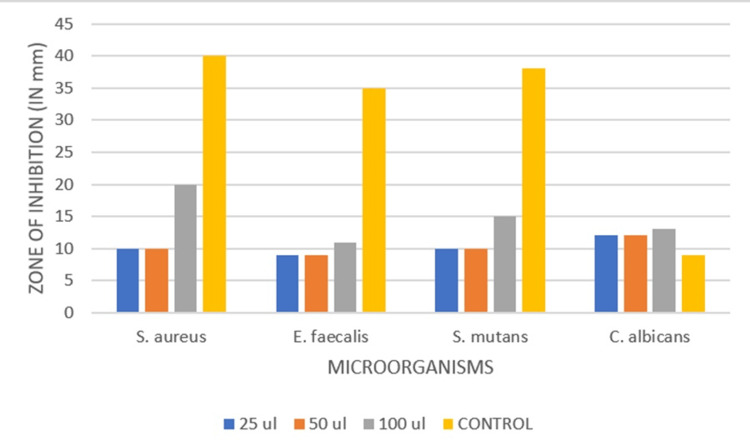
Antimicrobial effect of the nanoformulated mouthwash

For all microorganisms, the zone of inhibition at various concentrations ranged, as depicted in Figure 2.

Cytotoxicity test

The cytotoxic effect of the formulated mouthwash was assessed at different concentrations, i.e., at 5, 10, 20 and 40 g/mL, using the brine shrimp lethality test. The results revealed that at the lowest concentration tested (5 g/mL), both the mouthwashes exhibited the same percentage of survival of nauplii (90%). However, as the concentration increased, i.e., at 40 g/mL, the formulated mouthwash demonstrated 70% live nauplii, as indicated in Table 3.

**Table 3:** Cytotoxic effect of the chamomile tea and green tea-mediated zinc oxide nanoparticle (ZnONP)-based mouthwash using the brine shrimp lethality assay. The survival rates of nauplii were evaluated at various concentrations of plant extract and for control mouthwash in microliters at baseline, 24 hours and 48 hours. ZnONPs: zinc oxide nanoparticles

Plant extract (concentration)	5 ul	10 ul	20 ul	40 ul	Control (mouthwash)
Baseline	100%	100%	100%	100%	100%
24 hours	80%	70%	60%	80%	100%
48 hours	70%	50%	50%	70%	90%

## Discussion

In the present study, the agar well diffusion assay was employed to assess the antibacterial effectiveness of the ZnONPs and notable sensitivity against the following organisms: *E. faecalis, S. mutans, S. aureus and C. albicans*. These organisms were employed in the study as they are common inhabitants of the oral cavity, and assessment of the effectiveness of formulated mouthwash against these organisms will be beneficial for its use in patients affected with periodontal disease or caries. To evaluate toxicity, the brine shrimp lethality test was employed, indicating lower toxicity of the formulated mouthwash compared to commercial alternatives. This suggests its suitability for usage, implying that the herbal formulation could serve as a safer alternative, potentially contributing to overall well-being by minimising adverse reactions.

For *C. albicans*, even at lower concentrations, growth inhibition was observed for ZnONPs; hence, these could serve as alternatives for the treatment of ailments, such as oral candidiasis [15].

Concurrently, earlier research on the green synthesis of ZnOPs utilizing *Parthenium hysterophorus *demonstrated the highest zone of inhibition against *Enterobacter aerogenes* (36 mm), and it showed the least activity against *S. aureus *and *Bacillus*. Similarly, the antibacterial efficacy of lemongrass and mint-mediated ZnONPs was good against *E. faecalis and S. aureus*. The selection of chamomile tea and green tea focuses on the increasing interest in leveraging the inherent benefits of such plants, as corroborated by prior research [9].

A broth dilution study demonstrated ZnONP synthesis using *Cinnamomum tamala* leaf extract and the assessment of antibacterial efficacy against *S. aureus* [16]. In a comparable investigation, ZnONP synthesis exhibited promising activity against *Xanthomonas oryzae* [17]. ZnONPs synthesized from *Alstonia scholaris* have also been successfully synthesized, and these exhibited significant antimicrobial effects. Although there were no fatalities observed at greater dosages, this underscores that the cytotoxic effect is a dose-dependent effect [18]. Moreover, green-synthesised ZnONPs using C*oriander oleoresin* demonstrated promising outcomes in cytotoxicity assessments, showcasing reduced toxicity levels [19]. In addition, findings indicated that ZnONPs produced from *Abies webbiana* show less toxic effects comparatively. Consequently, this suggests the possibility of formulating various nanostructures with reduced amounts of these nanoparticles, offering a safer, eco-friendly and economically viable solution. The investigation into cytotoxic effects, conducted through a brine shrimp mortality assay involving ZnONPs derived from coffee beans and xylitol, demonstrated that at 80 g/mL, 80% nauplii remained alive. This finding correlates with the cytotoxic outcome observed in the current study regarding ZnONPs mediated by lemongrass and mint [18]. In addition, utilizing a co-precipitation technique, a combination of ammonium hydrogen phosphate and calcium chloride with sodium hydroxide was employed at pH 8, resulting in amorphous calcium phosphate (ACP) nanoparticles. The manufactured mouthwash was found to be similar to those of the previously synthesized ACP nanoparticles in terms of effectiveness against oral pathogens. Consequently, it is conceivable to consider that ACP nanoparticles have the potential to be effective against dental caries. In comparison to Oral-B and chlorhexidine, the created mouthwash offers several advantages; it is naturally made, less toxic and also cost-effective [20]. Dentists, especially periodontists who cater to patients with severe gum diseases, should be aware of the inherent risks of and the diversity of microbial colonisation in the oral cavity, in order to be able to provide proper treatment.

This research offers a thorough evaluation of both effectiveness against pathogens and biocompatibility, presenting convincing proof of the efficacy and safety of a new method for addressing oral infections utilizing herbal-mediated mouthwash. It offers inhibition of a broad spectrum of oral pathogens, along with limited cytotoxic responses, underscores the promise of these being used as alternatives.

Limitations

This is an in vitro study, which underscores the importance of conducting further research to validate the antimicrobial and cytotoxic properties of the mouthwash. Minimum inhibitory concentration (MIC) and minimum bactericidal concentration (MBC) can also be assessed in the future.

## Conclusions

The current study highlights the promising antimicrobial and cytotoxic activity of the synthesised formulation. This suggests that it could prove to be an effective alternative mouthwash for use in the oral cavity. Further detailed research on ZnONPs is essential because certain nanoparticles have shown adverse effects when they were used at higher concentrations. While the formulated mouthwash shows promise for therapeutic applications, further investigation is important in order to identify areas where it can be used in the treatment of gum health and to prevent oral diseases.
